# Relationships Between Task Constraints, Visual Constraints, Joint Coordination and Football-Specific Performance in Talented Youth Athletes: An Ecological Dynamics Approach

**DOI:** 10.1177/00315125231213124

**Published:** 2023-11-10

**Authors:** Pieter Heuvelmans, Stefano Di Paolo, Anne Benjaminse, Laura Bragonzoni, Alli Gokeler

**Affiliations:** 1 Exercise Science and Neuroscience Unit, Department of Exercise & Health, Faculty of Science, 26578 Paderborn University, Paderborn, Germany; 2 Department for Life Quality Studies, 9296 University of Bologna, Bologna, Italy; 3 Department of Human Movement Sciences, Faculty of Medical Sciences, 3647 University of Groningen, Groningen, the Netherlands; 4 School of Sport Studies, Hanze University Groningen, Groningen, the Netherlands

**Keywords:** ecological dynamics, football, constraints, joint coordination, performance

## Abstract

Individual performance in team sports is a multifactorial reflection of how well a player can cope and accomplish tasks in varied playing situations. Thus, performance analysis should not only focus on outcomes, but also on underlying mechanisms of those outcomes. We adopted principles of the ecological dynamics approach (EDA) to investigate the effect of introducing constraints on players’ joint coordination responses for a football-specific performance drill outcome. Seventeen talented youth football (soccer) players performed a football-specific drill under different conditions: basic constraints, additional defender dummies, stroboscopic glasses, and a combination of the latter two constraints. We recorded these players’ execution time, passing accuracy, and lower extremity joint kinematics. We calculated joint coordination for hip-knee, knee-ankle, and trunk-hip couplings. The added constraints negatively affected execution time and passing accuracy, and caused changes in joint coordination. Furthermore, we identified a relationship between execution time and joint coordination. This study serves as an example how the EDA can be adopted to investigate mechanisms that underlie individual performance in team sports.

## Introduction

Performance in team sports derives in part from a combination of physical and perceptual-cognitive factors governing an athlete’s ability to meet their goals in open-skill play (Davids et al., 2003, 2008). Varied playing situations arise from task, environment, and athlete-related constraints (Renshaw et al., 2010). Task constraints may include the athlete’s objective and any rules or objects that specify or constrain the athlete’s response dynamics, such as opponents blocking a desired passing direction. Environmental constraints may include features like the type of terrain and weather conditions. Athlete-related constraints may involve the individual’s own physical and mental characteristics (e.g., height, weight, limb length, and level of attention, motivation, or anxiety). These constraints serve as boundaries that shape an athlete’s self-organizing movement patterns (Renshaw et al., 2010). The effects of many constraints are mediated by the athlete’s ability to perceive them. For instance, while it has been demonstrated that visual–perceptual abilities are enhanced in more skilled versus less skilled athletes (Mann et al., 2007; Voss et al., 2010), stroboscopic vision has been shown to reduce sport performance, especially in skilled athletes (Beavan et al., 2021). These interaction effects highlight the importance of visual perception on sport performance.

One criticism of conventional methods of performance analysis is their focus on outcomes rather than underlying processes that produce those outcomes (Torrents & Balagué, 2006). In EDA, the focus is on understanding *how* players and teams regulate their sport performance (Seifert et al., 2017). The EDA integrates theories from the constraints-led approach, ecological psychology, and complex systems approach in neurobiology (Seifert et al., 2017). Hence, the EDA views players and teams as complex adaptive systems and recognizes that the relationship between movement coordination and performance may be non-linear and non-proportional (Seifert et al., 2017). To clarify, since every athlete has individual movement solutions intended to satisfy the constraints imposed on them (Renshaw et al., 2010), coordination between different joints or body segments may vary even when performing the same task (Weir et al., 2019). To best understand these complex interactions underlying sports performance, we must investigate how movements are coordinated and controlled within dynamic environments.

Studies that adopt the EDA preserve the athlete-environment relationship (Bolt et al., 2021). In the field, athletes are free from laboratory restrictions and can make sport-specific movements that should be the subject of study in performance analysis. Measuring “on the pitch,” however, complicates data interpretation with increased numbers of uncontrolled variables (Parada, 2018). Researchers should, therefore, be mindful of the steps that they take to preserve the athlete-environment interaction; they must take incremental steps, investigating only a small number of uncontrolled variables at once to facilitate interpretation of the data (Bolt et al., 2021). By applying the principles of the EDA on a football pitch, we hoped to examine how player movements are coordinated and how this performance is affected by additional constraints. Our aim in this study was to investigate the effect of additional task and athlete-constraints on the performance and joint coordination of talented youth football players during this single football-specific drill. Our secondary aim was to present a novel EDA-based method for investigating the mechanisms that underlie this performance. We hypothesised that players would demonstrate non-linear adaptations to the complexity of various constraints, and that subsequent joint coordination responses would explain differences in player performances.

## Method

### Participants

Seventeen talented male youth football (soccer) players (*M* age = 13.9. *SD* = .3 years; *M* height = 1.64, *SD* = .09 m; *M* weight = 50.9, *SD* = 7.4 kg) were recruited from the talent development program of the youth academy of a professional football club. All players were free from any neurological disease and/or visual impairment at the time of testing, and they had no history of serious lower extremity injury or surgery within the previous year, based on medical screening at the start of the season by medical staff of the youth academy. The players were all field players, at the competitive phase of the season (i.e., month of April) when their performance was most representative of their optimal abilities, and all players had trained 4–5 times per week. Prior to participants’ enrollment, we explained to them the purpose of the study and obtained both their informed written assent and the informed consent of their parents. The study and its procedures were approved by the ethics committee of Paderborn University, Paderborn, Germany.

### Procedures

**Figure 1.**
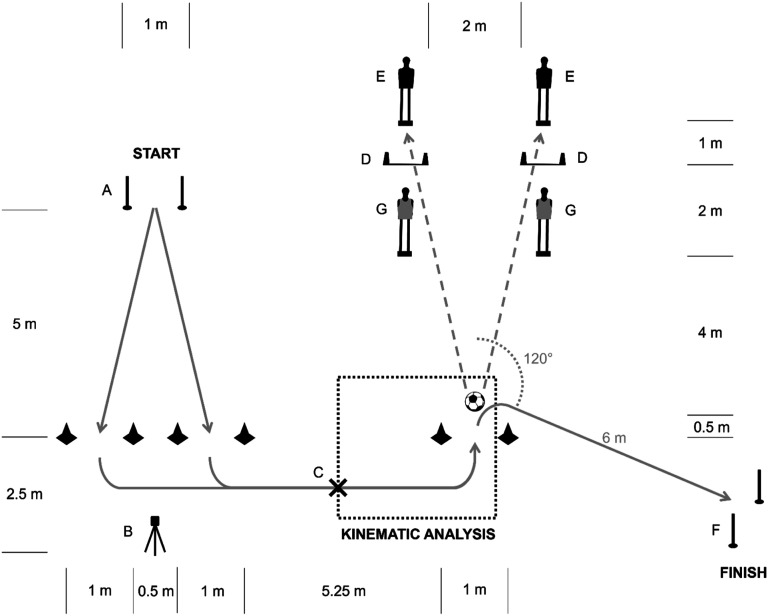
Illustration of the Football-Specific Drill. *Note*. After a visual start cue (i.e., arrow pointing to the left or right) from the Microgate LED indicator (B), the player sprinted from the timing gate at the start (A) through the left or the right set of cones. Following a 90-degree turn, the player then sprinted towards the football. When the player was at 3 m (C) from the football, one of the two SmartGoals (D) was manually activated by the same operator using a SmartRemote (SmartGoals B.V., the Netherlands). The SmartGoal that was activated indicated which direction the ball had to be passed to, hitting the target dummy (E) standing behind. The target dummy represented a teammate. After passing the ball, the player made a 120-degree turn and sprinted to the timing gate (F) at the finish line. Note: the vest-wearing dummies (G) representing opponents in front of the SmartGoals (D) were only included in conditions 2 and 4.

Players wore their own football shoes, and tests were performed in daylight on an artificial turf football pitch. We used the Microgate Witty SEM system (Microgate Srl, Italy) to indicate the player’s running direction at the beginning of the course by means of an LED indicator (Figure 1). We used a SmartGoals System (SmartGoals B.V., the Netherlands) to display the target goal. We used Senaptec Strobe glasses (SENAPTEC Inc., USA) to apply a perceptual constraint to the player. The lenses flickered between clear and opaque at 3 Hz. 3D lower extremity, and we collected trunk kinematics during each trial by means of wearable inertial sensors. Players wore a MVN Lycra suit (Xsens Technologies, the Netherlands) that holds 17 inertial sensors with an internal sample rate of 1000 Hz. The overall system output frequency is 240 Hz. We gathered anthropometric data from players and used it for the calibration of the inertial sensors, following manufacturer’s guidelines.

#### Football Drill

Players performed a football-specific drill on a course marked by cones, timing gates, SmartGoals, and dummies (Figure 1). Instructions for this drill included a demonstration run-through of the task and an explanation of the different conditions a player might face during the performance. Furthermore, players were instructed to (a) sprint at maximum speed and (b) score as many correct passes as possible, since both speed and accuracy are important aspects of football kick performance (Kellis & Katis, 2007). A correct pass was defined as hitting the target dummy behind the lit up SmartGoal. Players performed a standardized warm-up and were familiarised with the conditions and the course. Every player completed five trials of each condition in ascending order (non-randomized). Athletes had 30-second breaks between each trial and 2-minute breaks between each condition.

### Manipulated Constraints

The constraints or conditions under which players had to perform were as follows:

• Condition (1) Basic constraints: After a visual start cue (i.e., arrow pointing to the left or right) from a Microgate LED indicator, the player sprinted for 5 m, from the timing gate at the start through the left or the right set of cones. Following a 90-degree turn, the player then sprinted for approximately 7 m towards the football. When the player was at 3 m from the football, one of the two SmartGoals was manually activated by the same operator using a SmartRemote (SmartGoals B.V., the Netherlands). The SmartGoal that was activated indicated in which direction the ball had to be passed to hit the target dummy standing behind. The target dummy represented a teammate. After passing the ball, the player made a 120-degree turn and sprinted for 6 m to the timing gate at the finish line.
• Condition (2) Added task constraint: Dummies with orange vests representing defenders were placed in front of the SmartGoals (Figure 1). These obstacles reduced the opportunities for the ball trajectory to pass through the SmartGoals to hit the target dummy, and therefore represented an environmental task constraint for the player.
• Condition (3) Added athlete constraint: Players were instructed to perform the drill whilst wearing stroboscopic glasses. Hence, this constraint affected the athlete’s visual perception.
• Condition (4) Added task *and* athlete constraint: Included both the defender dummies and the stroboscopic glasses to simultaneously impose both an athlete constraint and a task constraint.

### Dependent Measures

The dependent variables we measured were:

• Execution time;
• Passing accuracy;
• Lower extremity and trunk kinematics.

Execution time was defined as the time elapsed from start to finish of the drill. Passing accuracy was calculated as the percentage of successful passes (i.e., hitting the dummy behind the lit up SmartGoal) for each condition. Lower extremity and trunk (thorax) kinematics were processed in a custom MATLAB script (The MathWorks, Natick, MA, US). A time-normalized window was defined for each trial by means of the center of mass trajectory in the anterior-posterior direction: the ultimate change of direction at 120° after the ball contact was considered as the final point of measurement (100%), while the penultimate change of direction was computed as the starting point of measurement (0%) (Figure 1). This window allowed the investigation of the footballers’ motion while approaching and kicking the ball and was not affected by the time or the number of steps taken. Inter-joint coordination was quantified through a modified vector coding technique with circular statistics (Chang et al., 2008).

### Player Joint Coordination Responses

We examined joint coordination for the following joint couplings:

• hip (+flexion/-extension) and knee (+flexion/-extension);
• knee (+flexion/-extension) and ankle (+flexion/-extension);
• trunk (+flexion/-extension) and hip (+flexion/-extension).

**Figure 2.**
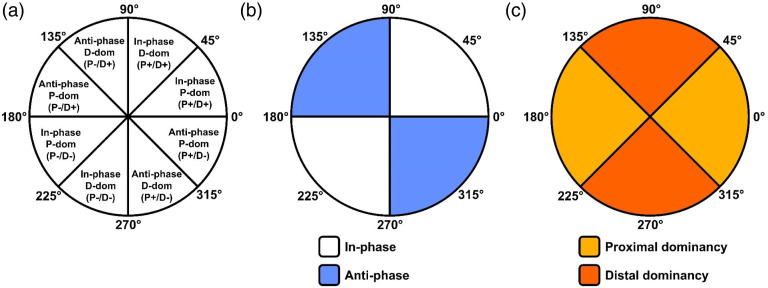
Schematic Polar Plots. *Note.* (A) The classification of coordination patterns based on the convention proposed by Needham et al. (2015); (B) the segments corresponding with in/anti-phase coordination; and (C) the segments corresponding with proximal/distal dominant coordination. P: proximal joint, D: distal joint, dom: dominancy, (+): flexion, (−): extension.

Sagittal plane joint coordination provides information about propulsion and deceleration strategies (Weir et al., 2019). We used a coordination pattern classification method (Figure 2(A)) to classify the joint coordination at each time point into anti or in-phase coordination with distal or proximal dominancy using the coupling angle obtained from vector coding (Needham et al., 2015). This classification method describes the relative motion between two joints and indicates which joint was the dominant mover at each time point. Furthermore, this method considers the direction of joint movement so that it can distinguish between, for instance, in-phase flexion and in-phase extension. As a result, we identified eight different coordination patterns (Figure 2(A)). To quantify the prevalence of each pattern, we calculated coordination pattern frequencies (CPFs) as the number of time points classified into a coordination pattern divided by the total number of time points. We performed vector coding and coordination pattern classification for each trial and averaged CPFs per player per condition to obtain coordination profiles. Coordination profiles were then averaged for each condition. As these coordination profiles provide detailed overviews of players’ movements, they are informative descriptors. However, to facilitate the interpretation of statistical analyses, we compared conditions on the sums of CPFs in anti-phase coordination (Figure 2(B)) and CPFs in distal dominancy (Figure 2(C)), respectively. We used a custom Python script in Spyder IDE (Python 3.9.9, Spyder 5.1.5) to conduct vector coding, coordination pattern classification, and CPF analyses.

### Statistical Analysis

We used the Shapiro-Wilk test to assess the normality of the data. We presented continuous variables as means and standard deviations and categorical variables as percentages of the total. We used a repeated measures analysis of variance (ANOVA) to assess any statistical differences between the four conditions on the measures of execution time, passing accuracy (i.e., 5/5 correct passes = 100% accuracy), anti-phase coordination, and distal dominant coordination. We set *p* < .05 to determine statistical significance. We used partial eta-squared for effect size and considered effect size categories as small, moderate, and large for values of .01, .06, .14, respectively (Cohen, 2013), and we reported effect size with Cohen’s d for multiple comparisons (small, moderate, and large effects for d values of .2, .5, .8, respectively). We investigated post-hoc comparisons among the single conditions through t-tests with Bonferroni corrections for multiple comparisons.

We also computed a stepwise linear regression model to investigate the association between joint coordination (hip-knee, knee-ankle, and trunk-hip distal dominancy and anti-phase coordination as independent variables) and performance data (execution time or passing accuracy as dependent variable). We reported effect sizes of the interactions with R^2^-adjusted and f^2^ values (small, moderate, and large effect for f^2^ values of .02, .15, .35). All statistical analyses were conducted in MATLAB.

Sample size was estimated according to previous literature (Besier et al., 2001; Heiderscheit et al., 2002; Seay et al., 2011). In particular, Seay et al. (2011) reported an effect size (Cohen’s d) of .34 for sagittal plane joint coordination analysis. We calculated a power analysis, using an ANOVA repeated measures with within-between factor interaction in G*Power (v3.1, Brunsbüttel, Germany), assuming a conservative effect size of .31, statistical power of 80%, and an alpha of .05, we found that a minimum of 16 participants was required.

## Results

**Figure 3.**
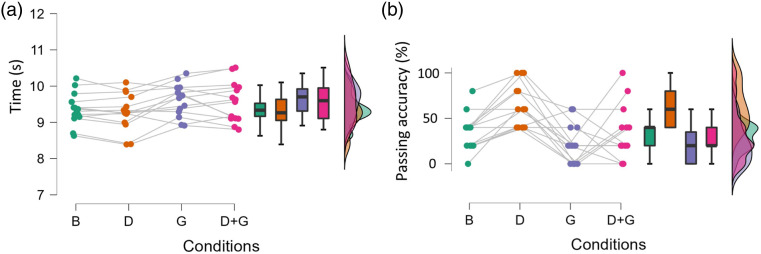
Distributions of Execution Time (A) and Passing Accuracy (5/5 passes = 100%, (B) per Condition with Connected Dots Representing Player Means. *Note*. B: basic constraints, D: defender dummies, G: stroboscopic glasses.

Three players were excluded from further analysis due to their left-leg dominance. Therefore, we conducted final analyses on 14 players. There was a statistically significant difference in execution time between the conditions (F(3,39) = 7.17, *p* < .001, η^2^*p* = .36) (Table 1, Figure 3(A)). Specifically, condition 2 (*M*_execution time_ = 9.3, *SD* = .5 seconds) was significantly faster than condition 3 (*M*_execution time_ = 9.6, *SD* = .4 seconds, d = .67, *p* = .002) and condition 4 (*M*_execution time_ = 9.6, *SD* = .6 s, d = .56, *p* = .010). There was also a statistically significant difference in passing accuracy between conditions (F(3,39) = 8.87, *p* < .001, η^2^*p* = .41) (Figure 3(B)), with accuracy in condition 2 (*M*_accuracy_ = 64.3, *SD* = 24.8%) significantly higher than condition 1 (*M*_accuracy_ = 34.3, *SD* = 19.9%, d = 1.25, *p* = .005), condition 3 (*M*_accuracy_ = 21.4, *SD* = 21.4%, d = 1.78, *p* < .001), and condition 4 (*M*_accuracy_ *=* 32.9, *SD* = 30.0%, d = 1.30, *p* = .004).

**Table 1.** Performance and Joint Coordination in the Different Constraint Conditions.

	Basic constraints	Dummies	Glasses	Dummies + glasses	*p*-value	η^2^p
Performance						
Execution time (s)	9.4 (.4)	9.3 (.5)	9.6 (.4)	9.6 (.6)	**< .001**	**.36**
Passing accuracy (%)	34.3 (19.9)	64.3 (23.8)	21.4 (21.4)	32.9 (30)	**< .001**	**.41**
Joint coordination (%)						
Hip-knee anti-phase	41.5 (8.8)	42.5 (7.4)	38.5 (3.8)	36.5 (6)	**.027**	**.21**
Hip-knee distal dominancy	71.7 (5.1)	71 (7.4)	67.4 (5.3)	69.5 (4.9)	.071	.16
Knee-ankle anti-phase	41 (5.8)	40.9 (7.6)	40.8 (7.1)	42.1 (7.9)	0.9	.02
Knee-ankle distal dominancy	33.6 (6.5)	35.2 (7.3)	36.3 (5.7)	34.1 (4.3)	.322	.09
Trunk-hip anti-phase	40 (7.6)	40.6 (6.2)	39.5 (7.7)	39.7 (7.2)	.866	.018
Trunk-hip distal dominancy	77.9 (4.9)	77.8 (5.9)	79.7 (4.7)	78.8 (3.8)	.554	.052

Note. Values are mean (standard deviation). Bold values represent statistically significant differences among the four conditions according to the repeated-measures ANOVA.

**Figure 4.**
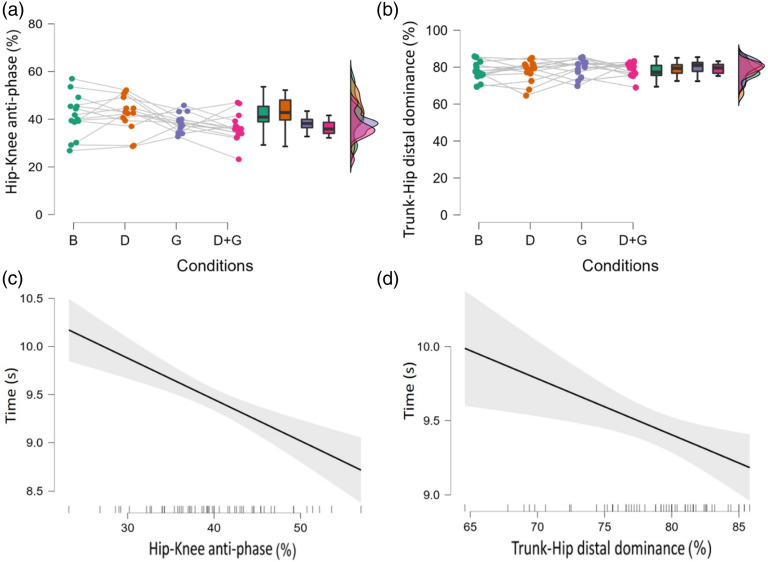
Distributions of hip-knee anti-phase coordination (A) and trunk-hip distal dominant coordination (B) per condition with connected dots representing player means. Stepwise linear regression analysis between execution time and hip-knee anti-phase coordination (C) and trunk-hip distal dominant coordination (D), respectively. *Note*. The explained variance in the regression model was *R*^2^ = .30, R^2^_Adjusted_ = .28 (*p* < .001). B: basic constraints, D: defender dummies, G: stroboscopic glasses.

There was a statistically significant condition difference in hip-knee anti-phase coordination (F(3,39) = 3.4, *p* = .027, η^2^*p* = .21) (Table 1, Figure 4(A)), with hip-knee anti-phase coordination in condition 2 (*M*_hip-knee anti-phase coordination_ = 42.53, *SD* = 7.41%) significantly higher (d = .89, *p* = .041) than condition 4 (*M* = 36.53, *SD* = 6.01%). Stepwise linear regression analysis identified a statistically significant relationship between execution time and hip-knee anti-phase coordination (Figure 4(C)) and trunk-hip distal dominant coordination (Figure 4(D)), respectively (*p* < .001, R^2^_Adjusted_ = .28, f^2^ = .46).

## Discussion

In this study we set out to investigate (a) the performance and joint coordination of talented youth football (soccer) players during a football-specific drill and (b) the effect of introducing additional constraints. Three main findings emerged. First, the performance measures, execution time and passing accuracy, were affected by the added constraints. Second, there were constraints-related changes in joint coordination (Appendix 1). Third, there was a significant relationship between execution time and hip-knee anti-phase and trunk-hip distal dominant coordination.

The players were instructed to complete the football-specific drill as fast as possible. Such a requirement is common in football training and many agility drills focus on speed. High intra-player performance variability was observed for execution time. The main trend was an individual increase in execution time from condition 2 to conditions 3 and 4 (Figure 3(A)). These observations were expected, as they represent intra-individual adaptations to the increased complexity imposed by the additional constraints. We also included ball passing accuracy as another football-specific performance measure. Only a small number of players showed high passing accuracy, with average passing accuracy below 40% in three conditions (Table 1, Figure 3(B)). Previous investigators have identified a trade-off between accuracy and velocity in kicking performance (Kellis & Katis, 2007; van den Tillaar & Fuglstad, 2017). This finding suggests that most players prioritized speed over accuracy, although we hypothesised that passing accuracy would increase if players were given more repetitions to learn and adapt their movement strategy. However, in real-life playing situations (i.e., a match), players are usually not offered multiple repetitions to optimize their motor strategy for successful performance. Therefore, sport-specific drills that introduce constraints with a limited number of attempts are likely to be effective in distinguishing players that adapt quickly from those who adapt slower (Supplementary Material).

Passing accuracy decreased with the introduction of the stroboscopic glasses, whilst average execution time increased (Table 1; Figure 3). Thus, the visual perturbation was sufficient to reduce overall performance. Training with stroboscopic devices is apt to improve perceptual-cognitive skills, which in turn transfers to enhanced sporting performance (Singh et al., 2021; Wilkins & Appelbaum, 2020; Wilkins & Gray, 2015; Wohl et al., 2021). We confirmed the strong interaction effect between visual perturbation and players’ coordination and performance. We conducted a single measurement. Future studies should consider the effect of added constraints after a period of visual perturbation training.

An interesting secondary finding from this study was the differences in performance measures among the conditions. Although condition 1 was used to represent baseline performance, execution time was lowest and passing accuracy was highest in condition 2. Since condition 2 introduced defender dummies which reduced the opportunities for ball trajectory in passing, we had expected instead an increase in execution time and/or decrease in passing accuracy. Two inferences may be drawn from these findings. On one hand, since conditions were non-randomized, a learning effect might have occurred, but, on the other, players may have had more focus and/or motivation for passing accuracy when the task complexity more closely resembled actual play, increasing sport-specific affordances to better perceive the task.

Hip-knee coordination showed a constraints-related change at the group level: average anti-phase coordination dropped by 6% in condition 4 compared to condition 2 (Table 1; Figure 4(A)). In other words, players moved their hip and knee more in-phase when they were simultaneously subjected to the stroboscopic glasses and the defender dummies, compared to when they only faced the dummies. Interestingly, a stepwise linear regression identified that hip-knee anti-phase coordination was significantly associated with execution time. Together with trunk-hip distal dominant coordination, hip-knee anti-phase coordination could explain 28% of the variance in execution time (Figure 4). These findings are exemplary first attempts at uncovering the mechanisms that underlie performance, by linking different types of joint coordination with a specific performance measure. Future studies that adopt the EDA may further contribute to understanding these mechanisms. Future investigators should study smaller windows of analysis for specific movements (e.g., braking, turning, kicking) to improve the resolution of coordination analysis.

The presence of defender dummies (conditions 2 and 4) and stroboscopic glasses (conditions 3 and 4) required a higher cognitive effort than the basic constraints (condition 1): the dummies narrowed the space for passing the ball and forced a specific trajectory, while the stroboscopic glasses reduced the players’ vision and their spatial perception (Beavan et al., 2021). The introduction of stroboscopic glasses (condition 3) coincided with a drop in average passing accuracy as well as an increase in execution time at the group level. However, intra-individually, some players managed to maintain or even increase their own performance (Figure 3). These results suggest that the incremental introduction of more demanding constraints may induce non-linear changes in motor control that are difficult to detect at the group level but which can affect individual performance, both positively and negatively. Previous investigators suggested an interaction between task complexity and joint coordination variability (Weir et al., 2019), and this interaction might ultimately affect movement efficiency and injury risk (Hamill et al., 2012).

### Limitations and Directions for Further Research

The present study had limitations to acknowledge. First, the football-specific drill (Figure 1) has not been previously validated. However, this drill included many aspects of game-like scenarios: visual information, decision-making, team-mate and opponent (dummies) factors, ball-kicking, and changes of direction. Second, the experimental setup favored right-side dominant players, since players had to make a left turn prior to kicking. Unfortunately, due to the exclusion of three left-dominant players, our sample size was below the target number set with power analysis; also, it was not possible to inspect the variability of individual responses through a model including IDs of the players as a random effect. Additionally, apart from statistical power associated with this small sample size, generalization from this small number of talented youth athletes to other populations is limited. Future investigators using similar drills should therefore design mirrored drills to accommodate both right and left-legged players equally. Third, the non-random order of conditions may have induced a learning effect. However, this possibility was not confirmed by statistical analysis. We assessed joint coordination for sagittal plane motion only, because of its importance in propulsion and braking. However, the frontal and transverse planes of motion may hold important information as well. Individual adaptations or compensatory movement strategies have potential implications for injury risk, and future investigators might explore joint coordination in the other planes of motion, using the methodology presented here.

## Conclusions

Our study was the first to propose and demonstrate an EDA based kinematical assessment of football players’ agility performance (Bolt et al., 2021). In particular, ours was the first study to investigate the effect of cognitive and physical constraints on players’ inter-joint coordination and performance during a sport-specific drill. Our findings have several practical implications. First, cognitively demanding agility drills seem to be effective in differentiating player performance, and they may be used for performance evaluation for talent selection and development. Importantly, however, our participant sample was relatively young. In this age group, the executive functions may not have yet been fully developed (Davidson et al., 2006). As such, a low-performing player from this demographic group may not only benefit from physical training, but may also require cognitive training and/or additional time to develop. A second practical implication concerns the observation that introducing new constraints to a drill (e.g., obstacles, rules) will likely affect players differently in accordance with the motor strategies players adopt. Since these adaptations are highly individualized, coaches/trainers should be cautious about interpreting constraints-induced player changes at a group level. Monitoring players individually may help in performance evaluation and injury risk assessment.

## Supplemental Material

Supplemental Material - Relationships Between Task Constraints, Visual Constraints, Joint Coordination and Football-Specific Performance in Talented Youth Athletes: An Ecological Dynamics Approach Click here for additional data file. Supplemental Material for Relationships Between Task Constraints, Visual Constraints, Joint Coordination and Football-Specific Performance in Talented Youth Athletes: An Ecological Dynamics Approach by Pieter Heuvelmans, Stefano Di Paolo, Anne Benjaminse, Laura Bragonzoni, and Alli Gokeler in Perceptual and Motor Skills

## Appendix 1

Coordination Profiles for Hip-Knee, Knee-Ankle, and Trunk-Hip Sagittal Plane Coordination Per Condition. *Note.* The bars in the polar plots represent the mean coordination pattern frequencies (CPFs), error bars indicate the pooled standard deviations. B: basic constraints, D: defender dummies, G: stroboscopic glasses.
