# Supplementary material for: Relationships Between Task Constraints, Visual Constraints, Joint Coordination and Football-Specific Performance in Talented Youth Athletes: An Ecological Dynamics Approach
Source: Percept Mot Skills. 2023 Nov 10;131(1):161–76. doi: 10.1177/00315125231213124 (PMC10863366; doi:10.1177/00315125231213124)
Supplement: Supplemental Material - Relationships Between Task Constraints, Visual Constraints, Joint Coordination and Football-Specific Performance in Talented Youth Athletes: An Ecological Dynamics Approach [file sj-pdf-1-pms-10.1177_00315125231213124.pdf]

Supplementary

<!--Col Count:5-->CV	Basic	Dummies	Glasses	Dummies + Glasses
Performance				
Execution time (s)	4.3	5.4	4.2	6.3
Passing accuracy (%)	58.0	37.0	100.0	91.2
Joint coordination				
Hip-Knee anti-phase	21.2	17.4	9.9	16.4
Hip-Knee distal dominance	7.1	10.4	7.9	7.1
Knee-Ankle anti-phase	14.1	18.6	17.4	18.8
Knee-Ankle distal dominance	19.3	20.7	15.7	12.6
Trunk-Hip anti-phase	19.0	15.3	19.5	18.1
Trunk-Hip distal dominance	6.3	7.6	5.9	4.8

Note. Coefficient of Variation (CV) for Performance and Joint Coordination in the Different Constraint Conditions.
